# The Case for Brachytherapy: Why It Deserves a Renaissance

**DOI:** 10.1016/j.adro.2020.10.018

**Published:** 2020-11-06

**Authors:** Vonetta M. Williams, Jenna M. Kahn, Nikhil G. Thaker, Sushil Beriwal, Paul L. Nguyen, Douglas Arthur, Daniel Petereit, Brandon A. Dyer

**Affiliations:** a Department of Radiation Oncology, University of Washington, Seattle, Washington; b Department of Radiation Oncology, Oregon Health & Science University, Portland, Oregon; c Department of Radiation Oncology, Arizona Oncology, Tucson, Arizona; d Department of Radiation Oncology, UPMC Hillman Cancer Center, Pittsburgh, Pennsylvania; e Department of Radiation Oncology, Dana-Farber/Harvard Cancer Center, Boston, Massachusetts; f Department of Radiation Oncology, Virginia Commonwealth University, Richmond, Virginia; g Department of Radiation Oncology, Monument Health Cancer Care Institute, Rapid City, South Dakota

## Abstract

The recent global events related to the coronavirus disease of 2019 pandemic have significantly changed the medical landscape and led to a shift in oncologic treatment perspectives. There is a renewed focus on preserving treatment outcomes while maintaining medical accessibility and decreasing medical resource utilization. Brachytherapy, which is a vital part of the treatment course of many cancers (particularly prostate and gynecologic cancers), has the ability to deliver hypofractionated radiation and thus shorten treatment time. Studies in the early 2000s demonstrated a decline in brachytherapy usage despite data showing equivalent or even superior treatment outcomes for brachytherapy in disease sites, such as the prostate and cervix. However, newer data suggest that this trend may be reversing. The renewed call for shorter radiation courses based on data showing equivalent outcomes will likely establish hypofractionated radiation as the standard of care across multiple disease sites. With shifting reimbursement, brachytherapy represents the pinnacle in hypofractionated, conformal radiation therapy, and with extensive long-term data in support of the treatment modality brachytherapy is primed for a renaissance.

## Introduction

Given recent global events related to the coronavirus disease of 2019 (COVID-19) pandemic, the medical landscape and oncologic treatment perspectives have significantly shifted. Oncologic physicians are increasingly focused on maintaining equipoise of treatment outcome and medical accessibility with decreasing medical resource utilization. In support of these measures, radiation oncologists have used a variety of temporizing measures, including hormone therapy measures (eg, breast, endometrial, and prostate cancer), treatment delays (where appropriate), and hypofractionation across all disease sites.^1^, ^2^, ^3^, ^4^, ^5^ For breast, prostate, and gynecologic malignancies, low-dose rate (LDR) and high-dose-rate (HDR) brachytherapy represent the pinnacle of hypofractionated, conformal radiation therapy. Previously, studies showed a decline in both gynecologic^6^^,^^7^ and prostate^8^ brachytherapy despite data showing superior treatment outcomes. However, newer data suggest that the declining utilization rates may be reversing.^9^^,^^10^ Brachytherapy treatment approaches are well tolerated, safe, effective, and cost-effective. As radiation oncologists and patients move forward, brachytherapy represents an often underused and effective treatment modality.

## Gynecologic Brachytherapy

Gynecologic brachytherapy is a vital and irreplaceable component of definitive and adjuvant treatment for gynecologic malignancies. Multiple studies have demonstrated the efficacy of brachytherapy to treatment outcomes in cervical and uterine oncologic malignancies.^11^, ^12^, ^13^, ^14^, ^15^, ^16^, ^17^, ^18^, ^19^ Unfortunately, gynecologic brachytherapy utilization has declined in parallel with the clinical implementation of intensity modulated radiation therapy (IMRT) and volumetric modulated arc therapy.^6^^,^^7^ Furthermore, attempts to replace brachytherapy with external beam treatment approaches have been unsuccessful. Notably, a recent phase 2 study of patients with predominantly locally advanced cervical cancer examined the feasibility of using a SABR boost as an alternative option to brachytherapy for medically unfit patients or those who refuse brachytherapy.^20^ The study was closed early owing to high toxicity rates, including death due to complications of therapy. The cervical cancer clinical practice guidelines by the American Society for Radiation Oncology state that either SABR or IMRT are only suitable replacements for brachytherapy when considered for patients refusing or ineligible for brachytherapy.^21^

Modern HDR brachytherapy is a form of hypofractionated, conformal therapy commonly delivered in 4 to 5 treatments for cervical cancer.^22^ However, there are 2- and 3-fraction regimens^23^^,^^24^ that have been used more in resource-poor settings that can be used to preserve resources in these times, decreasing the treatment time so that curative treatment can be delivered faster. Complex interstitial cases are often done in the operating room in the modern era, but gynecologic brachytherapy procedures can be safely delivered without use of operating room time in an HDR suite without the need for anesthesia or through the use of moderate sedation for interstitial cases.

Advances in imaging technology, such as magnetic resonance imaging, allow for adaptive image guided brachytherapy (IGBT) with simultaneous dose escalation to tumor targets and sparing of organs at risk. Compared with point-based brachytherapy planning, volumetric-based planning using IGBT has demonstrated improved tumor control and significantly reduced toxicity.^25^, ^26^, ^27^ Additionally, a cost-utility analysis of IGBT showed that magnetic resonance imaging has the potential to decrease health care costs compared with 2-dimensional or computed tomography–guided brachytherapy through reduced costs from cancer recurrence and treatment toxicity.^28^

## Breast Brachytherapy

Partial breast irradiation (PBI) has demonstrated comparable treatment outcomes to whole breast irradiation with regard to local tumor control, toxicity, and cosmetic outcomes.^29^, ^30^, ^31^, ^32^, ^33^ Initially, accelerated PBI provided a method to shorten typical 5- to 6-week standard fractionation radiation courses to 5 days. The recent publication of the UK Fast Forward study offers an even faster external beam option for the delivery of radiation to the breast.^34^ However, hypofractionation is still underused in the United States.^35^ Therefore, brachytherapy remains a viable, short treatment option with new data exploring noninvasive techniques and even shorter treatment regimens.^36^^,^^37^

Early data for breast brachytherapy delivered in 1 to 4 fractions have demonstrated excellent local tumor control and cosmetic outcomes.^37^^,^^38^ The phase 2 Triumph-T trial showed excellent local tumor control (albeit with a short median follow up) and breast cosmesis using a 3-fraction breast brachytherapy technique, and a similar 4-fraction regimen had excellent cosmesis with no locoregional recurrences at 6 years.^37^^,^^39^ Furthermore, in elderly patients, single-fraction regimens have also demonstrated excellent oncologic outcomes,^40^ and a recent study comparing PBI with PBI + hormone therapy or hormone therapy alone in women age >70 years with low-risk, hormone-positive, early stage breast cancer demonstrated that PBI was superior when compliance with hormone therapy was poor,^41^ and tested compliance interventions have demonstrated no improvement.^42^^,^^43^

Therefore, even with the likely adoption of shorter external beam radiation treatment regimens, breast brachytherapy remains an excellent option for women and provides good local control and cosmetic outcomes.

## Prostate brachytherapy

Prostate brachytherapy results in excellent treatment and toxicity outcomes has a short overall treatment time (OTT), and is more cost effective than other radiation treatment options. Prostate brachytherapy (HDR or LDR) is considered equivalent to radical prostatectomy and external beam radiation for the treatment of prostate cancer and can be completed in 1 (LDR) or several (HDR) implantations.^44^^,^^45^ The use of either LDR or HDR prostate brachytherapy decreases OTT compared with external beam standard fractionation and some hypofractionation schemes when used as a boost.^2^ As monotherapy, HDR and LDR approaches have a shorter OTT than SABR, which is typically delivered in 5 to 7 every-other-day fractions.^46^

Brachytherapy as monotherapy is appropriate for patients with low-risk or favorable intermediate-risk disease or as a boost in patients with unfavorable intermediate- and high-risk disease. When used as a boost for patients with unfavorable to high-risk disease, recent data from 2 prospective randomized trials have shown that brachytherapy significantly prolongs biochemical progression-free survival by >50% compared with dose-escalated external beam radiation.^47^^,^^48^ Furthermore, retrospective data also suggest that brachytherapy used as monotherapy for low-risk disease can prolong biochemical progression-free survival compared with either surgery or external beam radiation.^45^ In addition, the median cost of prostate cancer therapy has been shown to be less with brachytherapy compared with either SABR, IMRT, or proton therapy.^49^ A 2013 study by Hayes et al found that brachytherapy was the most effective and least costly initial treatment option for men with low-risk prostate cancer, including men who chose active surveillance.^50^ Fortunately, although older data suggested that treatment with prostate brachytherapy was declining,^8^ this trend appears to reversing.^10^

Prostate brachytherapy is also useful in the setting of isolated intraprostatic recurrence after definitive treatment with radiation. A recent phase 2 trial, as well as several retrospective studies, demonstrated excellent rates of cancer-free and biochemical recurrence-free survival with brachytherapy and had acceptable, predominantly grades 1 and 2 gastrointestinal and genitourinary toxicity.^51^, ^52^, ^53^ Compared with other local salvage techniques, such as prostatectomy, high-frequency ultrasound, or cryotherapy, prostate brachytherapy has similar rates of biochemical control at 5 years with lower toxicity rates, such as incontinence and bladder neck stricture.^54^ Prostate brachytherapy remains a viable treatment option for patients, provides excellent outcomes with acceptable toxicity, and is cost effective.

## Economic Considerations

The use of hypofractionation in the United States has been increasing, leading to a decline in radiation oncology departmental revenue through reduced episodic fee-for-service reimbursement.^55^^,^^56^ This trend coincided with a period of transition from volume- to value-based care. During this period, the total proportion of U.S. health care payments tied to quality- and cost-focused alternative payment models (APMs) increased from 23% in 2015 to 34% in 2017.^57^ The shift to value-based care was further accentuated by the recent Radiation Oncology APM (RO-APM) proposal from the Centers for Medicare and Medicaid Services in 2019.^58^

COVID-19 has since accelerated the transition to extreme hypofractionation, including stereotactic radiation therapy and brachytherapy. After COVID-19, we anticipate the continued use of shorter treatment schedules and modalities that minimize patient exposure to high-cost hospital resources, postoperative care, or hospitalization. Brachytherapy is well-positioned to capitalize on these changes given its high value proposition. Most brachytherapy treatments can be delivered with minimal resources,^2^ lower fully loaded treatment delivery costs via time-driven activity-based costing analyses,^59^^,^^60^ or in alternative locations, such as ambulatory surgery or freestanding centers.^61^ As a low-cost modality,^62^ brachytherapy can be associated with less patient coinsurance and copayment for patients who may be facing unemployment or reduced income, as well as loss of health insurance coverage. Despite these benefits, reduced physician reimbursement for brachytherapy has exacerbated a decline in revenues for practices that are already affected during the pandemic.^63^ This places radiation oncology practices at a further financial risk in an already high fixed-cost business.

The adoption of the RO-APM may improve financial stability by providing episodic payments for disease site-specific radiation oncology care. These payments would be tied to average episode reimbursements rather than the volume or modality of service. This APM redesign appropriately attempts to incentivize shorter courses of low-cost, high-quality treatment (ie, brachytherapy). This change would also protect physicians from uncontrollable downside risks, such as from COVID-19, and provide financially stable payments to practices.

However, despite these theoretical benefits, several key changes are necessary to the RO-APM to ensure sustainability of and access to radiation oncology care in the United States. A practice’s bundled reimbursement in the RO-APM will be closely tied to its historical reimbursements per episode of care. Practices that were early adopters of hypofractionation and high users of cost-effective treatments, such as brachytherapy (ie, efficient practices), will receive lower reimbursements than practices that have been slow adopters of hypofractionation or who have not used cost-effective modalities (ie, inefficient practices).^64^ The RO-APM also does not account for the cost of episodes of care that require combination modality therapies, including brachytherapy as a boost, and inadvertently incorporates palliative care episodes in the calculation of bundled rates. Solutions exist that can align incentives in the RO-APM toward high-value cancer care, including brachytherapy without unfairly penalizing efficient practices, which is a win for patients, providers, and society as a whole.

## Conclusions

Brachytherapy is vital and irreplaceable for gynecologic malignancies, and results in excellent treatment and toxicity outcomes for breast and prostate malignancies. Brachytherapy is value-based and cost effective. The utilization of brachytherapy declined in the early 2000s, and has been associated with a decrease in resident brachytherapy caseload. The decline in residency brachytherapy training has been identified as a barrier to achieving brachytherapy competence and clinical independence.^65^ In an effort to combat the decline in brachytherapy, some resident training centers have instituted brachytherapy simulation workshops to improve resident brachytherapy training,^65^^,^^66^ and the American Brachytherapy Society has called for expanded training opportunities.^67^ The American Brachytherapy Society initiated a 10-year strategic program to address the declining rates of brachytherapy utilization, referred to as 300 in 10. The goal is to train 30 competent brachytherapists per year over 10 years through a multifaceted approach that includes developing a national brachytherapy curriculum, simulation-based medical education, 2-month fellowships for senior-level residents, a certification process, and maintenance of certification.^68^

Given preexisting inclinations for shorter radiation courses, a new radiation oncology normalcy will likely establish hypofractionated radiation as the standard of care across multiple disease sites. With shifting reimbursement, brachytherapy represents the pinnacle in hypofractionated, conformal radiation therapy, and with extensive long-term data in support of the treatment modality brachytherapy is primed for a renaissance.
